# Predicting Nonalcoholic Fatty Liver Disease through a Panel of Plasma Biomarkers and MicroRNAs in Female West Virginia Population

**DOI:** 10.3390/ijms21186698

**Published:** 2020-09-13

**Authors:** Sneha S. Pillai, Hari Vishal Lakhani, Mishghan Zehra, Jiayan Wang, Anum Dilip, Nitin Puri, Kathleen O’Hanlon, Komal Sodhi

**Affiliations:** 1 Departments of Surgery and Biomedical Sciences, Marshall University Joan C. Edwards School of Medicine, Huntington, WV 25701, USA; pillais@marshall.edu (S.S.P.); lakhani@marshall.edu (H.V.L.); humayun@marshall.edu (M.Z.); wangji@marshall.edu (J.W.); anum.dilip@hotmail.com (A.D.); 2 Departments of Biomedical Sciences and Medical Education, Marshall University Joan C. Edwards School of Medicine, Huntington, WV 25701, USA; purin@marshall.edu; 3 Departments of Family Medicine, Marshall University Joan C. Edwards School of Medicine, Huntington, WV 25701, USA; kohanlon@marshall.edu

**Keywords:** nonalcoholic fatty liver disease, nonalcoholic steatohepatitis, obese, diabetes, biomarker, miRNA

## Abstract

(1) Background: Nonalcoholic fatty liver disease (NAFLD) is primarily characterized by the presence of fatty liver, hepatic inflammation and fibrogenesis eventually leading to nonalcoholic steatohepatitis (NASH) or cirrhosis. Obesity and diabetes are common risk factors associated with the development and progression of NAFLD, with one of the highest prevalence of these diseased conditions in the West Virginia population. Currently, the diagnosis of NAFLD is limited to radiologic studies and biopsies, which are not cost-effective and highly invasive. Hence, this study aimed to develop a panel and assess the progressive levels of circulatory biomarkers and miRNA expression in patients at risk for progression to NASH to allow early intervention strategies. (2) Methods: In total, 62 female patients were enrolled and blood samples were collected after 8–10 h of fasting. Computed tomography was performed on abdomen/pelvis following IV contrast administration. The patients were divided into the following groups: Healthy subjects with normal BMI and normal fasting blood glucose (Control, n = 20), Obese with high BMI and normal fasting blood glucose (Obese, n = 20) and Obese with high fasting blood glucose (Obese + DM, n = 22). Based on findings from CT, another subset was created from Obese + DM group with patients who showed signs of fatty liver infiltration (Obese + DM(FI), n = 10). ELISA was performed for measurement of plasma biomarkers and RT-PCR was performed for circulating miRNA expression. (3) Results: Our results show significantly increased levels of plasma IL-6, Leptin and FABP-1, while significantly decreased level of adiponectin in Obese, Obese + DM and Obese + DM(FI) group, as compared to healthy controls. The level of CK-18 was significantly increased in Obese + DM(FI) group as compared to control. Subsequently, the expression of miR-122, miR-34a, miR-375, miR-16 and miR-21 was significantly increased in Obese + DM and Obese + DM(FI) group as compared to healthy control. Our results also show distinct correlation of IL-6, FABP-1 and adiponectin levels with the expression of miRNAs in relation to the extent of NAFLD progression. (4) Conclusion: Our results support the clinical application of these biomarkers and miRNAs in monitoring the progression of NAFLD, suggesting a more advanced diagnostic potential of this panel than conventional methods. This panel may provide an appropriate method for early prognosis and management of NAFLD and subsequent adverse hepatic pathophysiology, potentially reducing the disease burden on the West Virginia population.

## 1. Introduction

Nonalcoholic fatty liver disease (NAFLD) is a heterogenous spectrum of diseases progressing from simple steatosis to nonalcoholic steatohepatitis (NASH) with/without fibrosis, cirrhosis and hepatocellular carcinoma (HCC), occurring in a dysmetabolic milieu [1,2]. It is rapidly becoming a major global health problem due to marked lifestyle changes, especially in western countries, with the rising prevalence closely mirroring the epidemic of obesity and Type II Diabetes Mellitus (T2DM). In the US, about 85 million people are afflicted with NAFLD and about 20–30% of these are estimated to have NASH with an expectation of exponential rise in NASH by 2025, to close to 43 million patients [3]. According to the 2018 West Virginia Behavioral Risk Factor Surveillance System Report, released by the West Virginia Department of Health and Human Resources, the prevalence of obesity and diabetes in West Virginia is around 37.7% (highest in the nation) and 15.0% (second highest nationally), respectively. The highest incidence of acute hepatitis B infection and acute hepatitis C infection in the US is also reported in West Virginia [4,5]. Studies have shown that hepatitis, in combination with obesity and insulin resistance, contributes to the disease progression of NAFLD in affected population [4,5].

Obesity has been recognized as a major underlying factor for the pathogenesis of several diseases, including metabolic syndrome, T2DM, hypertension and atherosclerosis. Growing evidence suggests that oxidative stress acts as the critical factor linking obesity with its associated co-morbidities [6,7]. Insulin resistance, which causes liver steatosis with increased hepatic lipogenesis and impaired free fatty acid degradation, sensitizes the liver to induce inflammation and cell death that promotes oxidative stress, culminating in NAFLD and fibrosis [8,9]. Increased intrahepatic levels of fatty acid induces oxidative metabolism, causing an increase in mitochondrial β-oxidation, peroxisomal β-oxidation. This, in turn, augments ROS production and inflammatory recruitment of cytokines such as IL-6 and TNF-α, which ultimately leads to the activation of apoptotic signaling pathways in liver [10,11]. Innate immune signaling and ROS signaling act synergistically during NAFLD to cause reprogramming of hepatic lipid metabolism, changes in insulin sensitivity and modulation of inflammation [8,12]. Furthermore, the adipokines released from the adipose tissue function in an autocrine and paracrine manner to mediate the release of macrophage chemokines and cytokines that further exacerbates the pathology of NAFLD progression [13,14].

Apart from these well-established mechanisms of hepatic fibrosis and disease progression, cumulative clinical and experimental evidence present in the literature suggests a role of cardiotonic steroids in mediating prooxidant and profibrotic effects in hepatic tissue [15,16,17,18,19,20,21,22]. While the hepatic tissue is a primary site for the maintenance of cholesterol homeostasis [23], studies have suggested an important role of cardiotonic steroids in regulation of cholesterol biosynthesis [24,25]. Therefore, the mechanistic action of cardiotonic steroids may further stimulate the synthesis of cholesterol in NAFLD, which may lead to the activation of hepatic stellate cells, substantially aggravating hepatic fibrosis or lipid accumulation [26,27,28,29,30] and redox inflammatory pathways [31,32,33,34]. The cumulative line of evidence also suggests the role of cardiotonic steroids in the activation of Na/K-ATPase signaling [35,36,37,38]. Na/K-ATPase signaling has been extensively shown to have signaling and scaffolding functions, distinct from its cellular ion pumping function [39]. Several studies have shown that cardiotonic steroids mediate signal transduction through Na/K-ATPase signaling, activating pathways associated with tissue fibrosis [15,19,22,40]. Previous in vivo studies have also demonstrated morphological evidence of steatohepatitis, induced by the cardiotonic steroid mediated activation of Na/K-ATPase signaling [40], along with alteration of hepatic lipid accumulation markers, fibrosis, inflammatory markers and mitochondrial fatty acid oxidation markers [40]. Since the activation of Na/K-ATPase signaling is mediated by cardiotonic steroid [15,41,42], the further downstream activation of this signaling cascade has also been shown to increase Homeostatic Model Assessment of Insulin Resistance (HOMA-IR) levels and blood glucose tolerance in high fat diet fed mice [43], suggesting an association of cardiotonic steroids with diabetes. Hence, cardiotonic steroids might be one of the possible mechanisms that may aggravate NAFLD.

MicroRNAs (miRNAs) are small non-coding RNAs that act as evolutionally conserved epigenetic regulators, affecting many physiological processes by involving in post-transcriptional regulation of gene expression [44]. Differential expression of miRNAs has been previously reported to play important roles in clinical and experimental NAFLD [44]. This is mainly through the regulation of altered lipid and glucose metabolism, oxidative stress, inflammation and pathways of hepatocellular survival and proliferation [45,46]. The miRNA signature of NAFLD has been extensively studied before, exploring the role of miRNAs in the various stages of disease progression [44,47]. As extracellular RNAs are typically short-lived due to ubiquitous RNase activity, short sequences of circulating endogenous miRNAs could be exploited as attractive candidate biomarkers for precise profiling of the different stages of the liver injury [48,49,50]. This can further enable clinicians for an early diagnosis and the clinical monitoring of the disease progression [44,51,52].

Even though liver biopsy is considered the gold standard for diagnosing and monitoring NASH [53], it is often deemed not appropriate for all patients with suspected NAFLD. Apart from poor cost effectiveness and being invasive, there remains a risk of serious complications, including pain at the biopsy site, serious bleeding and rarely death [54]. With an increasing number of patients developing NASH-related end-stage liver disease, the development and validation of non-invasive biomarkers that accurately diagnose and predict clinically relevant outcomes to monitor both NAFLD and fibrosis are critically needed. The findings from our laboratory previously demonstrated the correlation between obesity, insulin resistance and biomarkers associated with NASH in pediatric patients from WV [55]. Owing to the increasing burden of obesity, metabolic disease and poor health care system, the risk of developing NAFLD in WV population is very high, especially in the female population. Hence, based on a review of literature, the present study aimed to study a panel of plasma biomarkers and miRNAs in adult female population, as predictive markers of NAFLD in WV. Based on this panel of biomarker, clinicians can monitor disease progression in patients with NAFLD-associated risk factors which may result in earlier detection and prognosis of NASH. This panel of biomarker is specifically important in the WV population as it may allow for earlier intervention strategies, resulting in improved NAFLD or subsequent NASH associated mortality, improved health outcomes and an improved overall healthcare cost.

## 2. Results

### 2.1. Demographics Data and Lipid Profile of Patients in Comparison with Healthy Subjects

The population characteristics and demographic data are detailed in Table 1. There was no significant difference in age and blood pressure among all study groups; hence, these parameters were not the influencing factors in any of the subsequent findings. The average BMI of the obese (*p* < 0.01), Obese + DM (*p* < 0.01) and Obese + DM(FI) (*p* < 0.01) were found to be significantly high when compared to that of healthy control. The assessment of FBG clearly showed the diabetic status of the study population with significantly high levels of FBG in Obese + DM, with greatest increase noted in Obese + DM(FI) as compared to control and obese populations. The assessment of lipid profile in these subjects clearly indicated signs of dyslipidemia associated with obese and diabetic conditions (Table 1). Obese, Obese + DM and Obese + DM(FI) showed increased level of triglycerides, LDL, VLDL and total cholesterol levels when compared to healthy control (Table 1), whereas HDL level, the good cholesterol, was found to be significantly decreased in Obese + DM(FI) subjects in comparison with healthy control. Finally, a significantly high LDL/HDL ratio was noted in Obese and Obese + DM (FI) subjects as compared to controls (Table 1).

### 2.2. Assessment of Patients’ Clinical Profile in Comparison with Healthy Subjects

The albumin level was significantly decreased in Obese + DM (*p* < 0.05) as compared to obese, while further decrease was noted in Obese + DM (FI) (*p* < 0.01) when compared to control and obese subjects (*p* < 0.01) (Table 2). An increasing trend was observed in the activity of hepatotoxicity enzymes such as alkaline phosphatase and alanine transaminase (ALT) when progressing from obesity to diabetic conditions as evidenced by the significantly high value of these enzymes in Obese + DM (FI) group (*p* < 0.01) (Table 2). Bilirubin levels were significantly decreased in Obese + DM as compared to control. Even though no significant changes were observed in the level of aspartate aminotransferase (AST) and BUN, the level of creatinine was found to be significantly increased in Obese + DM (FI) population when compared to all other groups (Table 2). The summary of these findings from complete clinical profile (CCP) of the study population is presented in Table 2.

### 2.3. Evaluation of Plasma Biomarkers in Obese and Diabetic Patients Compared to Healthy Subjects

**Figure 1:**
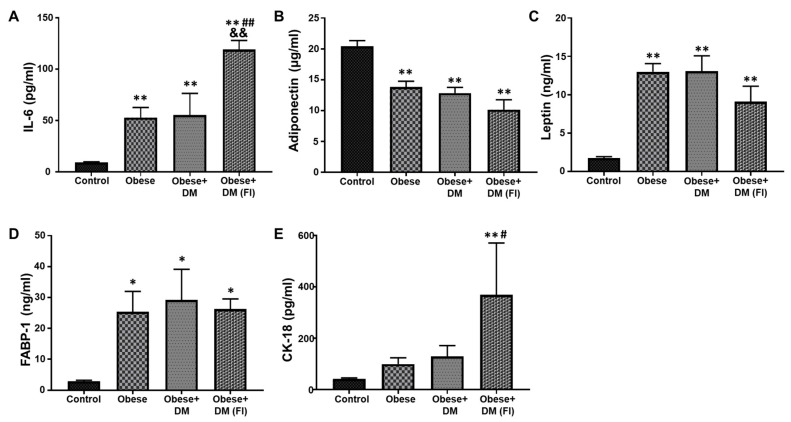
Quantitative analysis of plasma biomarkers. ELISA assay was performed to determine concentration of key plasma biomarkers in the patient population, which showed progressively altered levels under diseased condition. Plasma concentrations of: (**A**) IL-6; (**B**) adiponectin; (**C**) leptin; (**D**) FABP-1; and (**E**) CK-18. Control (n = 20), Obese (n = 20), Obese + DM (n = 12) and Obese + DM(FI) (n = 10). Values represent means ± SEM. * *p* < 0.05, ** *p* < 0.01 vs. Control; # *p* < 0.05, ## *p* < 0.01 vs. Obese, && *p* < 0.01 vs. Obese + DM.

The blood-based biomarkers can be useful to monitor, diagnose and classify the disease progression in NAFLD. In the present study, we analyzed a panel of important biomarkers that are reported to have significant effects on NAFLD progression. The plasma level of IL-6, an important marker for systemic inflammation, was significantly elevated in Obese and Obese + DM compared to healthy subjects (Figure 1A). Obese + DM (FI) showed a further significant increase in the level of IL-6 as compared to Control, Obese and Obese+ DM (Figure 1A). The level of adiponectin and leptin can be used to establish the role of adipose tissue dysfunction in the progression of NAFLD. Compared to healthy subjects, the level of adiponectin showed a significant decrease in Obese, Obese + DM and Obese + DM(FI) patients (Figure 1B). On the other hand, the levels of plasma leptin were significantly higher in Obese, Obese + DM and Obese + DM (FI), as compared to healthy subjects (Figure 1C). The circulating level of FABP-1, a protein mainly expressed in liver and secreted into circulation, was also measured in the plasma. Compared to healthy control, the level of FABP-1 was significantly increased in Obese, Obese + DM and Obese + DM(FI) subjects (Figure 1D). The evaluation of plasma CK-18, a marker of hepatic apoptosis, showed significantly higher levels in Obese + DM(FI) subjects as compared to other subjects (Figure 1E).

### 2.4. Evaluation of Circulating miRNAs in Obese and Diabetic Patients Compared to Healthy Subjects

**Figure 2:**
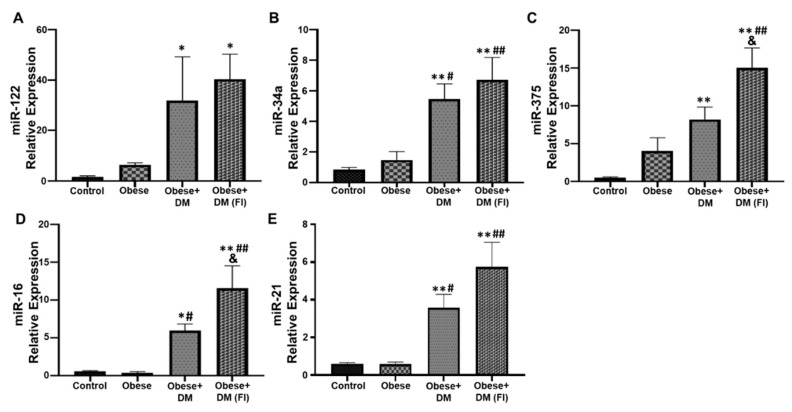
Assessment of circulating miRNAs expression. qRT-PCR was performed to determine expression of key circulating miRNAs in the patient population, which showed progressively altered levels under diseased condition. miRNA expression of circulating: (**A**) miR-122; (**B**) miR-34a; (**C**) miR-375; (**D**) miR-16; and (**E**) miR-21. Control (n = 20), Obese (n = 20), Obese + DM (n = 12) and Obese + DM(FI) (n = 10). Values represent means ± SEM. * *p* < 0.05, ** *p* < 0.01 vs. Control; # *p* < 0.05, ## *p* < 0.01 vs. Obese, & *p* < 0.05 vs. Obese + DM.

As circulating miRNAs play a significant role in the disease progression of NAFLD, the present study evaluated the relative expression of some important miRNAs in our subjects. miR-122 was significantly upregulated in Obese + DM and Obese + DM(FI) as compared to control and obese subjects (Figure 2A). In comparison with control, the expression of miR-34a was significantly high in Obese + DM and Obese + DM (FI) (Figure 2B). The relative expression of miR-34a was also elevated in Obese + DM and Obese + DM(FI) compared to Obese (Figure 2B). Similarly, the expression of miR-375 was found to be significantly up regulated in Obese + DM and Obese + DM(FI) as compared to control (Figure 2C). A further upregulation of miR-375 was observed in Obese + DM(FI) compared to Obese and Obese + DM. Apart from that, Obese + DM showed significant upregulation in the expression of miR-16, as compared to control and Obese group (Figure 2D). There was a further upregulation of miR-16 expression noted in Obese + DM(FI), compared to control, Obese and Obese + DM (Figure 2D). Next, we noted significant upregulation in the expression of miR-21 in Obese + DM and Obese + DM(FI) as compared to control and Obese subjects (Figure 2E).

### 2.5. Correlation Analysis and Diagnostic Performance of Plasma Cytokine Biomarkers and Circulating miRNAs in the Study Population

**Figure 3:**
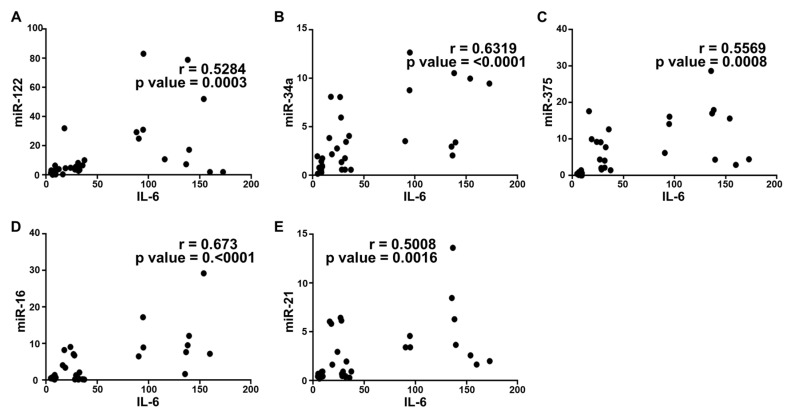
Correlation analysis of IL-6 with circulating miRNAs expression. Correlation was determined using Pearson’s r coefficient choosing two tailed *p*-value to demonstrate significance (alpha = 0.05). Scatter dot plot between IL-6 and the following: (**A**) miR-122; (**B**) miR-34a; (**C**) miR-375; (**D**) miR-16; and (**E**) miR-21. Each plot independently shows corresponding correlation coefficient (r value) and significance (*p* value).

**Figure 4:**
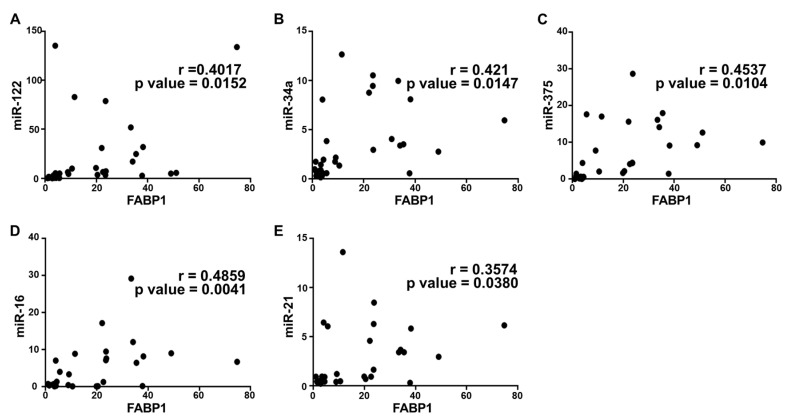
Correlation analysis of FABP-1 with circulating miRNAs expression. Correlation was determined using Pearson’s r coefficient choosing two tailed *p*-value to demonstrate significance (alpha = 0.05). Scatter dot plot between FABP-1 and the following: (**A**) miR-122; (**B**) miR-34a; (**C**) miR-375; (**D**) miR-16; and (**E**) miR-21. Each plot independently shows corresponding correlation coefficient (r value) and significance (*p* value).

**Figure 5:**
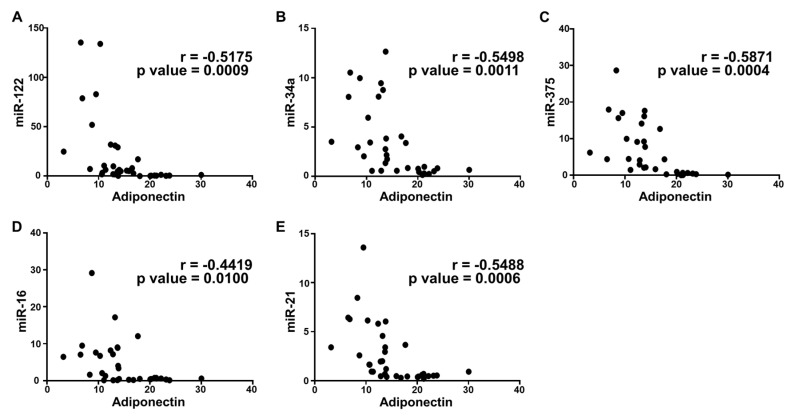
Correlation analysis of adiponectin with circulating miRNAs expression. Correlation was determined using Pearson’s r coefficient choosing two tailed *p*-value to demonstrate significance (alpha = 0.05). Scatter dot plot between adiponectin and the following: (**A**) miR-122; (**B**) miR-34a; (**C**) miR-375; (**D**) miR-16; and (**E**) miR-21. Each plot independently shows corresponding correlation coefficient (r value) and significance (*p* value). A negative correlation coefficient suggests an inverse correlation.

To further confirm the correlation between plasma cytokines and circulating miRNAs in the disease progression of NAFLD, we performed a statistical correlation analysis, where the extent of correlation was assessed in terms of Pearson’s r coefficient. Even though we performed the correlation analysis of all cytokines with each miRNA included in the study, three were found to have significant correlation with all microRNAs. Plasma IL-6 and FABP-1 showed a positive correlation with all miRNAs in the study population with different stages of metabolic dysregulation (Figure 3 and Figure 4). The comparison of mean plasma levels of IL-6 showed positive correlation with the expression of miR-122 (r = 0.5284) (Figure 3A), miR-34a (r = 0.6319) (Figure 3B), miR-375 (r = 0.5569) (Figure 3C), miR-16 (r = 0.673) (Figure 3D) and miR-21 (r = 0.5008) (Figure 3E). Similarly, there was a positive correlation in the mean plasma levels of FABP-1 and the expression of miR-122 (r =0.4017) (Figure 4A), miR-34a (r = 0.421) (Figure 4B), miR-375 (r = 0.4537) (Figure 4C), miR-16 (r = 0.4859) (Figure 4D) and miR-21 (r = 0.3574) (Figure 4E). Consequently, the plasma adiponectin levels showed a significant inverse correlation with the expression of miR-122 (r = −0.5175) (Figure 5A), miR-34a (−0.5498) (Figure 5B), miR-375 (−0.5871) (Figure 5C), miR-16 (−0.4419) (Figure 5D) and miR-21 (r = −0.5488) (Figure 5E), as the disease advances from obesity to diabetes causing fatty infiltration, which may further progress to severe form of NASH. We further estimated the diagnostic performance of the dysregulated plasma cytokines and circulating miRNAs in our patients’ cohort by determining the predictive value of these biomarkers. The performance of each plasma cytokine and circulating miRNA was considered good with approximate AUROC of >0.80 (Table 3). The optimal cut-off values were determined using Youden’s index. Our results show good overall diagnostic performance of these biomarkers with a significant *p*-value, which shows predictive efficacy of these biomarkers in NAFLD (Table 3).

## 3. Discussion

**Figure 6:**
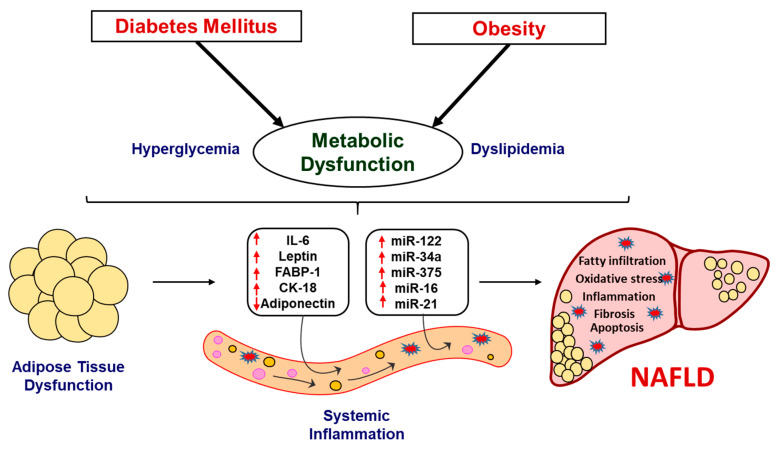
Schematic representation demonstrating the progression of NAFLD. The metabolic dysregulation in obese and associated diabetes in patients causes alteration in key systemic and liver enzymes. These pathological changes alter the systemic levels of key biomarkers and circulating miRNAs, which are involved in the regulation of hepatic function. The alteration in these biomarkers and miRNAs may lead to the development and progression of NASH. Hence, the utilization of panel of these specific biomarkers and miRNAs may allow tracking disease progression and, subsequently, early intervention strategies.

NAFLD is the most prevalent etiology of chronic liver disease worldwide that progress to NASH, which is characterized by the presence of lobular inflammation and hepatocyte ballooning, with or without fibrosis and may emerge as the leading reason of end-stage liver disease in the near future [53,56]. Giving importance to the growing prevalence of obesity, diabetes and associated complications that results in NAFLD and NASH in the West Virginian population, the present study demonstrates a panel of biomarkers and circulating miRNAs that can be used as predictive markers for NAFLD progression. Recently published studies have demonstrated the efficacy of several plasma cytokines and circulating miRNAs, independently, in a patient cohort, suggesting their role in NAFLD. These studies were inclusive of NAFLD and NASH patients, showing altered levels of biomarkers associated with inflammation, oxidative stress, fibrosis and apoptosis [56,57,58,59,60]. However, this study presents a panel of biomarkers with a unique combination of plasma cytokines and miRNAs, which, to the best of our knowledge, has not been established previously. Our results show altered levels of adiponectin, leptin, IL-6, FABP-1 and CK-18 and differential expression of important miRNAs such as miR-122, miR-34a, miR-375, miR-16 and miR-21, in obese and diabetic subjects. These alterations were noted to be exacerbated in patients with fatty infiltration of liver. The correlation of these biomarkers and miRNAs with the NAFLD progression reveal the usefulness of the present investigation in diagnosis and prediction of disease severity in NAFLD. Although our results are statistically significant and demonstrate translational applicability for the use of these biomarkers and miRNA in a clinical setting, there were several limitations of the study. These limitations include a small sample size of patients with “fatty liver infiltration” status, as well as limitation in obtaining liver biopsies for a rigorous assessment of NASH. In addition, this study does not include findings from the patients’ follow-up, which would potentially strengthen the predictive outcomes of the study. Furthermore, this study assessed only female population; hence, there were limitations in assessing gender-based differences in predicting the levels of these biomarkers and miRNAs. The overall study, summarized in a schematic diagram (Figure 6), demonstrate potential markers and their significance in early detection and prognosis of NAFLD and associated pathology.

Dyslipidemia is characterized by altered level of lipids, lipoproteins and impaired glucose tolerance, evidenced by the increased level of FBG, predisposes patients to various metabolic disorders including NAFLD [61,62]. Our results are in concordance, as the system lipid profile was severely altered in our obese and diabetic patients with or without fatty infiltration of liver. Previous studies have demonstrated altered secretion of various liver function enzymes into the circulation, in patients under different stages of NAFLD [63]. The enzymes such as alkaline phosphatase and ALT are highly specific to liver and are associated with hepatotoxicity, apoptosis, inflammation and other form of liver injury [63,64,65,66]. Our results are in concordance with previous findings, as these liver specific enzymes were significantly dysregulated in Obese + DM(FI) group. Hence, the findings in the present study confirms hepatotoxicity over the course of progression of NAFLD. Sustained inflammation due to metabolic abnormalities has been evidenced to play an important role in several processes critical to the development of obesity and diabetes, with a major adverse impact on key steps of fatty liver progression to fibrosis [67]. The localized adipose tissue inflammation propagates an overall systemic inflammation associated with the development of obesity-related comorbidities and further progress to NASH [68,69]. Various cytokines are reported to act as mediators of injury, inflammation, fibrosis and cirrhosis in NASH [70]. Cumulative evidence suggests that the systemic levels of IL-6, a cytokine with pleiotropic functions, are consistently increased in obesity, diabetes and eventually in NAFLD [71,72,73]. In agreement with previous studies, our results also show that the systemic levels of IL-6 were significantly increased in obese and diabetic conditions and even further increased in patients with fatty infiltration. Hence, the exacerbated systemic inflammation characterized by elevated circulating levels of cytokines and activation of pro-inflammatory signaling pathways during the NAFLD progression can be clearly evidenced from our results showing the progressive increase of IL-6 and thus can be used as a reliable plasma cytokine marker.

Growing evidence suggests that adipokine alterations, which occur during the expansion of adipose tissue under metabolic stress, contribute to the development and progression of NAFLD [74]. Among the major adipokines, adiponectin is highly abundant in serum and antagonizes excess lipid storage in the liver and protects from inflammation and fibrosis [75]. It exhibits anti-steatotic effect on the hepatocytes by increasing free fatty acid oxidation, decreasing gluconeogenesis, inhibiting de novo lipogenesis, and by protecting hepatocytes from apoptosis [76,77]. Evidence shows that adiponectin is secreted by adipose tissue in inverse proportion to the BMI and the levels of adiponectin are decreased in patients with obesity and related pathologies [78,79]. In accordance with these reports, our results also show a significant decrease in the level of adiponectin in patients with obesity and diabetes with or without fatty infiltration. However, leptin, an important adipokine having pro-inflammatory function, is reported to be secreted proportionally to the amount of white adipose mass, and thus circulating leptin levels reflect body energy stores and acute changes in caloric intake [80]. Leptin administration has been shown to enhance liver fibrosis by increasing the expression of procollagen-I, TGF*β*1 and smooth muscle actin and amplify inflammation by increasing the systemic levels of TNF-α in experimental models [81]. In line with these reports, our results show a significant increase in the circulating levels of leptin in patients with obesity and diabetes with or without fatty infiltration. The progressive changes in the level of adiponectin and leptin in obese and diabetic conditions that further leads to fatty infiltration shows their applicability as major adipokines in predicting the disease progression towards NAFLD.

Fatty acid-binding proteins (FABPs) secreted from liver coordinate lipid responses in hepatocytes and link various metabolic and inflammatory pathways which include fatty acid uptake, transport, oxidation, lipid synthesis and storage [82]. Cumulative evidence suggests the positive correlation of FABP-1 with obesity, diabetes and NASH [83,84]. In accordance with previous reports, our results showed increased levels of FABP-1 in patients with obesity and diabetes with or without fatty infiltration. Hence, the increased activity of FABP1 and the resulting enhanced intracellular trafficking of fatty acids in dyslipidemic condition may be shunting more detrimental fatty acids to storage and thereby promoting steatosis and NASH. In addition to abnormal fat metabolism, hepatocyte apoptosis also plays an important role in liver injury and thereby NAFLD progression. Evidence suggests that activated caspases and Bcl-2 family proteins induced hepatocyte apoptosis, caused by abnormalities in glucose and lipid metabolism, stimulate immune cells and hepatic stellate cells towards the development of fibrosis in the liver through the production of inflammasomes and cytokines [85]. The activation of apoptotic cascades lead to the proteolytic cleavage of CK-18, the major intermediate filament protein in the liver and the resulting CK-18 fragments are reported to be high in liver and plasma in NAFLD and NASH patients [86,87]. In agreement with these studies, our results show a significantly high level of plasma CK-18 in patients with obesity and diabetes having fatty infiltration. Our results point to the characteristic morphologic changes of apoptosis that are occurring in liver in accordance with fatty infiltration that further leads to NASH.

Recent studies have reported the role of various miRNAs as epigenetic modifiers, which can determine not only the early risk assessment but also the disease progression and prognosis in NAFLD and NASH [42,44]. Various circulating miRNAs have been reported to act as signaling molecules that can cause cell-to-cell communication, mainly between hepatocytes, hepatic stellate cells and adipocytes, which may result in fibrogenesis and liver damage progression that ultimately lead to NASH [48,56]. Based on a detailed literature review, we selected five miRNAs to investigate in our study population. miR-122 is a highly abundant liver-specific miRNA associated with cholesterol and hepatic free fatty acid oxidation and metabolism [88,89]. Multiple studies have shown that miR-122 is significantly increased in the pathogenesis of fatty infiltration and fibrotic changes associated with NAFLD progression, which can be positively correlated to serum CK-18 levels [50,90,91]. Evidence also suggests that elevated circulating levels of miR-122 can be positively associated with obesity and insulin resistance [89]. In accordance with these previously published reports, the present study also showed a significantly upregulated expression of miR-122 in response to obesity and diabetes progressing towards hepatic fatty infiltration and NAFLD. Another important miRNA, miR-34a, is a highly lipid responsive miRNA in the liver, modulating oxidative stress metabolism and apoptosis and is reported to be highly expressed in patients with diabetes, steatosis, NASH and in experimental models of NAFLD [92,93]. miR-34a is a well characterized regulator of sirtuin 1 (SIRT1) [49] and studies demonstrate that inhibition of miR-34a can ameliorate altered glucose metabolism, obesity and steatosis by restoring SIRT1 and peroxisome proliferator-activated receptor α (PPARα) signaling [94] and β-Klotho/Fibroblast growth factor-19 signaling [95,96]. In line with previous studies [97,98,99], our results also show a significantly upregulated expression of miR-34a in patients with obesity, diabetes and fatty infiltration, which can be used as a biomarker for disease progression. Similarly, miR-375, is a key regulator of glucose homeostasis, lipid metabolism, inflammatory response and insulin resistance under various metabolic stress conditions and is found to be upregulated in the circulation during obese, diabetic, simple steatosis and NASH [50,100,101]. The progressive increase in the expression of miR-375, as demonstrated in the present study, makes it a suitable candidate to predict the staging of NASH progression. Furthermore, miR-16 dysregulation promote liver fibrosis in hepatic stellate cells by upregulating guanine nucleotide-binding α-subunit 12 (Gα12) mediated pro-fibrogenic signaling that in turn stimulate hepatic stellate cells activation through autophagy-mediated breakthrough of lipid droplets and possess a positive effect on TGF-β/Smad signaling pathway [102,103,104]. As evident from previous reports and the results of our study, miR-16 can be effectively correlated with the severity of steatosis, fibrosis and NASH progression [49]. Studies depict that miR-21 promotes hepatic lipid accumulation and modulates extracellular signal-regulated kinase 1 (ERK1) signaling and epithelial-mesenchymal transition in liver fibrosis, and hence it is found to be upregulated in serum and hepatic tissues of individuals with different stages of NAFLD [105,106]. The significantly high expression of miR-21 expression in our study population with obesity and diabetes shows the trend towards fibrosis and NASH, which was confirmed by the highest expression in the population with fatty infiltration.

The dysregulation of these miRNAs over metabolic dysfunction in the different stages of NAFLD were further validated by performing correlation analysis with cytokine biomarkers. As miRNAs can regulate a wide spectrum of biological processes and metabolic homeostasis, the correlation analysis demonstrates the usefulness of these plasma biomarkers and miRNAs as a panel of predictive markers in NAFLD. Our results showed significant correlation of major biomarkers like IL-6, adiponectin and FABP-1 with all the miRNAs studied. This further confirms the coordinated interrelation between circulating miRNAs and signaling mediators involved in inflammatory and dyslipidemic pathways associated with NAFLD and that can be utilized for disease prediction, diagnosis and further treatment.

## 4. Material and Methods

### 4.1. Patients

In total, 62 female patients, visiting the Family Medicine Clinic at Joan C. Edwards School of Medicine, were enrolled in this study. This study randomly chose to utilize female subjects; however, future studies will also assess male patients as well as determine gender specific differences in the overall outcomes. Patients with any cardiovascular disease including hypertension, any hematological disorder, malignancy, trauma, autoimmune disease, acute or chronic kidney disease, any recent gastrointestinal surgery, pregnancy or age under 18 or over 60 were excluded from this study. Any patient with other hepatic disorders such drug-induced liver disease, alcoholic liver disease, viral hepatitis, schistosomiasis, primary biliary cirrhosis, sclerosing cholangitis, hemochromatosis, Wilson’s disease and biliary obstruction were also excluded from the study. To ensure an appropriate selection of patients eligible for the study, trained hospital personnel examined patients’ medical records with appropriate confidentiality measures and in compliance with HIPAA. The patients were selected according to the study’s inclusion criteria, which were based on their body mass index (BMI), fasting blood glucose (FBG) and age >18 or <60, with or without “fatty liver infiltration” status as noted on computed tomography (CT) scan. None of the participants who were enrolled in the study were on any antihypertensive medications, antibiotics, weight loss medications or any other medication for chronic disease, as described previously [107,108]. Based on their diabetic status, patients were only on glucose lowering drugs such as metformin, sulfonylureas or insulin, as long as they were on a stable dose 3 months prior to enrollment. Trained hospital personnel followed a standard protocol to measure the height, weight and waist circumference (midway from the lowest rib to the iliac crest to the nearest 0.1 cm) [109]. BMI was calculated by dividing weight (kg) by the square of height (m). Each patient was briefed about the use of the blood sample for this clinical study and each patient signed an informed consent. The latest diagnostic criteria by World Health Organization (WHO) on obesity classifies patients with a BMI of <25 kg/m^2^ as “normal”, while a BMI of >30 kg/m^2^ is classified as “obese”. Subsequently, WHO classifies FBG of <100 mg/dL as “normal”, while FBG of >126 mg/dL as “diabetic”. Hence, the patients were divided into three main groups according to their inclusion criteria which was based on their BMI and FBG levels: (I) healthy subjects with normal BMI (≤25 kg/m^2^) and normal FBG levels (≤100 mg/dL) (Control, n = 20); (II) patients with high BMI (≥30 kg/m^2^) but normal FBG levels (≤100 mg/dL) (Obese, n = 20); and (III) patients with high BMI (≥30 kg/m^2^) and high FBG levels (≥126 mg/dL), having clinical diagnosis of type II diabetes mellitus (Obese + DM, n = 22). Furthermore, abdominal computed tomography (CT) was performed, at the Radiology Department of Cabell Huntington Hospital, to determine any signs of liver steatosis. Findings from our CT scan showed 10 out of 22 Obese + DM patients had fatty liver infiltration. Hence, based on the findings of the CT scan, these patients were further divided in two subsets: Obese + DM (n = 12) and Obese + DM(FI) (n = 10). This research was approved by the Ethics Committee of the Cabell Huntington Hospital, West Virginia (Institutional Review Board No. 1168633, 19 April 2018). Written informed consent was obtained from all participants.

### 4.2. Blood Samples

Blood samples were obtained from eligible patients, after fasting for at least 8 h, by trained personnel who followed standard protocol to withdraw venous blood. Approximately 5 mL of blood were withdrawn from antecubital vein into the EDTA tubes. All blood samples were processed within 30 min of withdrawal by centrifugation at 4000 rpm for 10 min under temperature settings of 4 °C. Following centrifugation, plasma was separated from the blood and collected in appropriately labeled Eppendorf tubes. Plasma was further divided to make aliquots of each sample to avoid continuous freeze–thaw cycles. All samples were stored in −80 °C to be further used for measurement of biomarkers and miRNA expression. The laboratory at Cabell Huntington Hospital analyzed all blood samples for lipid panel, glucose levels and complete chemical profile (CCP) for the assessment of clinical parameters, which were later accessed in patients’ electronic health records.

### 4.3. Computed Tomography (CT) Imaging

All CT scans were performed at the Radiology Department at Cabell Huntington Hospital. Standard protocol was followed for CT imaging by a trained CT technologist (American Registry of Radiologic Technologists (ARRT) certified) under the supervision of a senior radiologist. CT examination of the abdomen and pelvis was obtained following low-osmolar IV contrast administration. The IV contrast enhancement was performed as directed by the supervising radiologist using appropriate injection protocols and in accordance with the ACR-SPR Practice Guideline for the Use of Intravascular Contrast Media and the ACR Manual on Contrast media. The patient was positioned in the center within the gantry and the scan was performed from top of the liver to pubic symphysis, while the patient was instructed to hold the breath at end of inspiration. The scan delay was set at 55–60 s. The CT images obtained in PACS were interpreted and signed by the radiologist.

### 4.4. Assessment of Plasma Biomarkers

Plasma samples stored in −80 °C were used for the assessment of biomarker levels by Enzyme-Linked Immunosorbent Assays (ELISA). The manufacturer’s protocol was followed for each of the following biomarkers using commercially available ELISA kits: adiponectin (Abcam, Cambridge, MA, USA), fatty acid binding protein 1 (FABP-1) (Abcam, Cambridge, MA, USA), cytokeratin-18 (CK-18) (Abcam, Cambridge, MA, USA), interleukin-6 (IL-6) (Abcam, Cambridge, MA, USA) and leptin (Abcam, Cambridge, MA, USA). All assays were performed using samples in duplicate to minimize statistical error. Briefly, the assay was performed in a 96-well plate in accordance with manufacturer’s protocol, and, at the end of the assay, the plate was read at 450-nm wavelength, in BioTek ELx800 Absorbance Reader. A standard curve was plotted for each biomarker and the concentrations were calculated using the resulting equation from the line of best fit.

### 4.5. Extraction of miRNA and Real Time Reverse Transcriptase-Polymerase Chain Reaction (RT-PCR)

RNA was extracted from plasma samples using the miRNeasy Serum/Plasma Kit (Qiagen, Hilden, Germany) according to manufacturer’s protocol. The quantity and quality of RNA was determined by 260:280 ratio using NanoDrop Analyzer (Thermo Scientific, Waltham, MA, USA). Following RNA extraction, miRCURY LNA RT Kit (Qiagen, Hilden, Germany) was used for our RT reactions, to synthesize cDNA, with 50 ng of total RNA for each reaction, as described previously [109,110]. To normalize the miRNA expression, an internal control, a synthetic spike-in, was used. Next, miRNA specific primers were used, combined with SYBR Green Master Mix at a final volume of 10 μL, to perform RT-PCR reactions in a 96-well plate. Three technical replicates were used per sample to ensure accuracy in the RT-PCR amplification data which was run on a 7500 Fast Real Time PCR System (Applied Biosystems, Foster City, CA, USA). The comparative threshold cycle (Ct) method was used to calculate the fold amplification as specified by the manufacturer. The following is the sequence of human miRNA primers used for our RT-PCR reactions: hsa-miR-122–3p: 5’AACGCCAUUAUCACACUAAAUA; hsa-miR-34a-3p: 5’CAAUCAGCAAGUAUACUGCCCU; has-miR-375–3p: 5’UUUGUUCGUUCGGCUCGCGUGA; hsa-miR-16–5p: 5’UAGCAGCACGUAAAUAUUGGCG; has-miR-21–3p: 5’CAACACCAGUCGAUGGGCUGU

### 4.6. Statistical Analysis

We ensured that our study was designed, conducted, recorded, analyzed and interpreted in an unbiased way and our results are reproducible before publication. All statistical analysis was performed using GraphPad Prism 7.0. Bartlett’s test was applied for each biomarker to guarantee equal variance. All data were tested for normality and then subjected to parametric analysis. One-way ANOVA was performed prior to comparison of individual groups and the Tukey’s post-hoc t-test was performed for multiple comparison. All significance was assigned at *p* < 0.05 or *p* < 0.01 for confidence interval of 95% or 99%, respectively. Each bar represents values as means ± standard error of mean (SEM). Correlation analysis was performed between biomarkers and each miRNA by plotting a scatter dot plot. A Pearson’s r coefficient was used to determine the extent of correlation using a 95% confidence interval and choosing two-tailed *p*-value to determine significance (alpha = 0.05).

The accuracy and the diagnostic performance of the plasma cytokines and circulating miRNAs was assessed by determining the area under the receiver operating characteristic (AUROC) curve analysis. The optimal cut-off for the best sensitivity and specificity was assessed by Youden’s Index. The 95% confidence interval was used for all AUROC analysis.

## 5. Future Perspectives

The modern dietary habits, genetic, epigenetic and environmental factors play key roles in the development and progression of obesity associated metabolic disarray and eventually NAFLD. The growing prevalence of these pathologies in the western world explains the need to identify specific biomarkers, attempting to distinguish and diagnose different stages of NAFLD, especially in a state such as West Virginia, where the incidence of these diseases is high. The findings from the present study highlight the role of key cytokine biomarkers and miRNAs, show the progressive change in the advancement of obesity and diabetes towards hepatic fatty infiltration and NAFLD, and the significant correlation among them gives the possible spectrum of accurate diagnosis. Given the cost effectiveness and non-invasive methodology, this panel is important for future studies. This panel may assist healthcare providers to allow early intervention for NAFLD prior to the irreversible complications in the disease progression. Future studies will also assess the plasma levels of cardiotonic steroid, marinobufagenin (MBG), to establish a possible role in the disease progression of NAFLD or subsequent NASH. The possibility to assign a panel of miRNA expression along with circulating cytokines for NAFLD represents an innovative breakthrough for identifying new important diagnostic tools to tailor efficient therapeutic interventions and more accurate prognosis and open specific avenues for future research. As a result, this may lead to improved healthcare outcomes and alleviate disease burden resulting in overall decreased morbidity and mortality associated with it.

## Figures and Tables

**Table 1:** Summary of BMI, fasting blood glucose and lipid profile in study subjects.

Groups	Control	Obese	Obese + DM	Obese + DM (FI)
Number of Patients (n)	20	20	12	10
Age (years)	42.7 ± 1.5	46.3 ± 1.6	49.4 ± 3.6	51.3 ± 4.3
Systolic Blood Pressure (mmHg)	119.3 ±2.3	126.6 ± 2.4	122.8 ± 2.4	126.0 ± 4.6
Diastolic Blood Pressure (mmHg)	74.0 ± 1.9	81.3 ± 2.5	76.3 ± 1.5	77.3 ± 2.4
Body Mass Index (kg/m^2^)	22.7 ± 0.8	39.2 ± 1.9 **	42.7 ± 4.2 **	34.5 ± 2.8 **
Fasting Blood Glucose (mg/dL)	84.9 ± 1.3	89.4 ± 2.4	134.7 ± 12.4 *^,#^	175.2 ± 25.9 **^,##^
Triglycerides (mg/dL)	93.5 ± 8.3	109.3 ± 9.0	154.3 ± 19.6 *	142.6 ± 27.1
High Density Lipoproteins (mg/dL)	63.6 ± 3.4	54.6 ± 4.4	55.6 ± 3.8	43.0 ± 3.6 **
Low Density Lipoproteins (mg/Dl)	88.8 ± 3.9	121.1 ± 7.1 **	107.1 ± 10.57	117.9 ± 7.766 *
Very Low-Density Lipoproteins (mg/dL)	15.1 ± 2.9	18.6 ± 2.4	26.3 ± 2.7 *	28.7 ± 3.7 *
Total Cholesterol (mg/dL)	167.4 ± 5.6	192.0 ± 6.6	186.8 ± 13.5	201.8 ± 9.8 *
LDL/HDL Ratio	1.50 ± 0.17	2.44 ± 0.24 *	1.99 ± 0.22	2.71 ± 0.34 **

Characteristics of the patient population, enrolled in the study, demonstrating their obese and diabetic status based on BMI and FBG levels, respectively. The lipid panel studied in each patient in their respective groups, shows general altered lipid profile as the disease progresses from obesity and diabetes to fatty liver. Values represent means ± SEM. * *p* < 0.05, ** *p* < 0.01 vs. Control; # *p* < 0.05, ## *p* < 0.01 vs. Obese.

**Table 2:** Summary of complete chemical and liver profile in study subjects.

Groups	Control	Obese	Obese + DM	Obese + DM (FI)
Number of Patients (n)	20	20	12	10
Albumin (g/dL)	4.41 ± 0.09	4.53 ± 0.07	4.02 ± 0.09 ^#^	3.63 ± 0.21 **^,##^
Alkaline Phosphatase (U/L)	57.38 ± 3.48	71.08 ± 3.33	79.10 ± 7.31	95.60 ± 17.8 **
Alanine Aminotransferase (ALT) (U/L)	16.9 ± 1.1	28.0 ± 3.5	24.8 ± 2.5	48.8 ± 9.9 **^,##,&&^
Aspartate Transaminase (AST) (U/L)	20.08 ± 1.53	26.17 ± 3.64	19.80 ± 3.31	27.90 ± 7.79
Bilirubin, Total (mg/dL)	0.69 ± 0.07	0.49 ± 0.07	0.42 ± 0.04 *	0.58 ± 0.06
Blood Urea Nitrogen (BUN) (mg/dL)	12.08 ±0.78	11.93 ± 0.59	14.10 ± 0.99	12.50 ±1.2
Creatinine (mg/dL)	0.68 ± 0.03	0.74 ± 0.04	0.79 ± 0.03	0.89 ± 0.09 *

The clinical profile in study subjects demonstrating progressive dysregulation of key markers and enzymes altered under diseased states. Values represent means ± SEM. * *p* < 0.05, ** *p* < 0.01 vs. Control; # *p* < 0.05, ## *p* < 0.01 vs. Obese, && *p* < 0.01 vs. Obese + DM.

**Table 3:** Diagnostic performance of predictors of NAFLD assessed by ROC curve analysis.

Biomarkers	AUROC	95% CI	*p*-Value	Optimal Cut-Off
*Plasma Cytokines*				
IL-6	0.86	0.72; 1.00	0.0009	>18.95 pg/mL
Adiponectin	0.86	0.72; 1.01	0.0031	<15.01 μg/mL
Leptin	0.89	0.75; 1.04	0.01	>3.15 ng/mL
FABP-1	0.90	0.78; 1.02	0.0008	>4.08 ng/mL
CK-18	0.83	0.61; 1.04	0.0061	>48.65 pg/mL
*miRNAs*				
miR-122	0.85	0.69; 1.00	0.0041	>5.55
miR-34a	0.86	0.71; 1.02	0.0045	>1.98
miR-375	0.88	0.72; 1.03	0.0064	>2.90
miR-16	0.86	0.69; 1.03	0.007	>1.48
miR-21	0.83	0.65; 1.02	0.0105	>0.92

AUROC were calculated to determine optimal cut-off value using Youden’s Index for all plasma cytokines and circulating miRNAs. The 95% confidence interval (CI) is provided along with the *p*-value to demonstrate statistical significance for each plasma cytokine and circulating miRNA.
